# Transcriptome meta-analysis reveals a central role for sex steroids in the degeneration of hippocampal neurons in Alzheimer’s disease

**DOI:** 10.1186/1752-0509-7-51

**Published:** 2013-06-26

**Authors:** Jessica M Winkler, Howard S Fox

**Affiliations:** 1 Department of Pharmacology and Experimental Neuroscience, University of Nebraska Medical Center, Omaha, NE 68198, USA

**Keywords:** Androgen receptor, Estrogen receptor, Graph theory, Hippocampus, Neurodegeneration

## Abstract

**Background:**

Alzheimer’s disease is the most prevalent form of dementia. While a number of transcriptomic studies have been performed on the brains of Alzheimer’s specimens, no clear picture has emerged on the basis of neuronal transcriptional alterations linked to the disease. Therefore we performed a meta-analysis of studies comparing hippocampal neurons in Alzheimer’s disease to controls.

**Results:**

Homeostatic processes, encompassing control of gene expression, apoptosis, and protein synthesis, were identified as disrupted during Alzheimer’s disease. Focusing on the genes carrying out these functions, a protein-protein interaction network was produced for graph theory and cluster exploration. This approach identified the androgen and estrogen receptors as key components and regulators of the disrupted homeostatic processes.

**Conclusions:**

Our systems biology approach was able to identify the importance of the androgen and estrogen receptors in not only homeostatic cellular processes but also the role of other highly central genes in Alzheimer’s neuronal dysfunction. This is important due to the controversies and current work concerning hormone replacement therapy in postmenopausal women, and possibly men, as preventative approaches to ward off this neurodegenerative disorder.

## Background

Alzheimer’s disease (AD) is of high interest in neurodegenerative research because an increasing rate of occurrence and lack of effective treatment or prevention. By 2050, 1 in 85 people globally are predicted to suffer from AD. This increase is thought to be due to the elevation in life expectancy and the resulting growing elderly population [1]. Signs of AD first begin with problems in short term memory progressing to a loss of long term memory and body functions. AD progression continues with increasing loss of memory and faculties ending in death [2]. Brain pathologies, amyloid beta plaques, neurofibrillary tangles and a loss of synaptic connections contribute to the progression [3].

Many AD studies focus on the brain’s memory and learning specific area, the hippocampus. The hippocampus, which functions in consolidation of new memories, emotional responses, navigation, and spatial orientation, is affected early in AD. The functions of the hippocampus are progressively disrupted, and AD neuropathology can be prominent in the hippocampus [4]. In order to obtain molecular clues to the etiology and pathogenesis of AD, investigators have performed a number of gene expression profiling studies on the hippocampus of AD and control brains [5-13]. Yet synthesizing the information from these different studies has been problematic. One of the reasons for this is the variability that stems from numerous sources during biological experimentation. Four of these are prominent in such studies on AD: the use of post mortem human samples, potential differences in the brain regions and cellular composition of examined specimens, carrying out experimentation in different labs, and the generation of high density “omic” data.

While microarray analysis has grown in popularity since its introduction 20 years ago many limitations have been found in both array and protocol design, including batch effect, uniform hybridization conditions for the probes, and ratio compression. However being a rather mature technology such problems are well known and these effects can be taken into consideration in the data analysis methods.

In our study we utilized a meta-analysis technique. Meta-analysis refers to the statistical methods for combining data from similar biological studies. Microarray datasets are excellent meta-analysis candidates due to the high use and deposit in publically accessible data banks, complete with information on experimental conduct. Advantages of meta-analysis include an increase in precision due to increased effect size, control for between-study variation and overcoming bias of individual studies [14,15]. As different microarray platforms can have different limitations as well as in some cases yield different results, we designed our study design by choosing independent experiments that use the same platform. By doing so the technical specifics and probe sequences are the identical. Furthermore we used a non-parametric permutation test based on ranks instead of gene expression values themselves to capture statistically significant genes that change between the conditions [15-17].

These differentially changed genes were then assessed through a systems biology approach. Systems biology can have many applications; we refer to its use to obtain biological information through utilizing whole-genome transcriptome analysis to assess networks and their interactions through the use of computational methods. Here we report that a meta-analysis of specific microarray datasets investigating AD, followed by a systems biology approach, yields unique insights into AD etiopathogenesis.

## Results

### Dataset gathering, ensemble mapping and expression analysis

Studies on gene array expression profiling for AD have examined different stages of disease and different areas of the brain. One important aspect of such studies on the brain is that the dissected tissue contains neurons, as well as glia (astrocytes, oligodendrocytes and microglia) and other cell types such as endothelial cells and pericytes. Gene expression in cell-types of interest within specific regions of the brain can be studied through microdissection, usually through laser capture. The functional pathology in AD is primarily linked to the neurons themselves. Given the predominance of the hippocampus in the initiation and progression of the disease, we searched the Gene Expression Omnibus (GEO) for transcriptional profiling studies on hippocampus in AD in which neurons had been microdissected. Two such studies were identified in which neurons were laser captured from the CA1 region of the hippocampus (Table 1) [5-7], totaling 17 arrays from AD cases and 21 arrays from controls. Both studies utilized Affymetrix Human Genome U133 Plus 2.0 GeneChip arrays; no such studies have been performed on other platforms or other technologies such as SAGE or RNA-Seq.

**Table 1:** **GEO datasets used in the meta**-**analysis**

**GEO dataset**	**ADC source of cases**	**Group description**	**Braak stage**	**Age**	**Brief summary of findings**
GSE28146	University of Kentucky	Control	2.3 ± 0.4	85.3 ± 2.7	Down regulation of molecules that stabilize ryanodine receptor Ca2+ release
		Severe AD	5.8 ± 0.2	84.0 ± 4.0	Up regulation of vasculature development
GSE5281	Arizona, Duke University, Washington University	Not AD	1.2 ± 0.1	79.6 ± 2.6	Mitochondrial and electron transport dysfunction
		Definite AD	5.3 ± 0.2	77.8 ± 1.8	Expression changes of metabolism-related genes

*ADC* Alzheimer’s Disease Center. *Braak Stage* Neuropathological staging according to [18]. Values for Braak stage and Age are mean ± S.E.M.

Most microarray analysis occurs by mapping to platform specific “probe” or “probe set” IDs with manufacturer-supplied annotations. This approach poses three problems: errors and irrelevancies, multiple IDs for a single gene, and combining multiple microarray platforms. While the latter does not apply to this study, the others pose a serious problem to accurate data analysis. The Affymetrix platform utilizes multiple probes assembled into probe sets to define the expression of genes. Using the GeneMapper program we deconvoluted the probe sets and remapped the probes to a singular identifier for known genes updated to our current understanding of the human genome. Fitting our criteria, we choose Ensemble Gene (ENSG) IDs for re-mapping individual probes from the probe sets to gene IDs, and reassembling the correctly mapping probes into new sets resulting in probe sets corresponding to 20,172 ENSG IDs.

**Figure 1:**
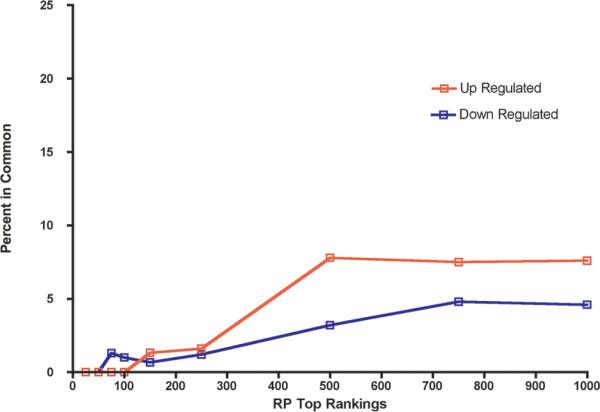
**Correspondence at the Top plot.** The x-axis is top ranking genes based on rank product. The y-axis is the percentage of genes in common among between the two studies at each level of ranking, for the upregulated (red) and down regulated (blue) genes.

After remapping, we then evaluated the presence of differentially expressed genes (DEGs) between AD and controls in each separate experiment. We utilized Rank Product analysis, a non-parametric method based on geometric mean of fold changes, to produce a fold-change, statistics and a ranking for each ID. Importantly Rank Product is efficacious for both single studies as well as meta-analysis. We then used the rankings to construct a Correspondence At the Top (CAT) plot, measuring proportions of DEGs in common between the two studies [19]. The CAT plot revealed that there was very little in common between the two studies (Figure 1), unfortunately not an infrequent finding, especially when each study itself has a limited sample size. Therefore we combined data for meta-analysis, again using Rank Product analysis, in order to obtain a clearer picture of the transcriptomic changes in neurons in AD.

### Meta-analysis and bioinformatics of hippocampal neurons in AD reveals dysfunction in homeostatic processes

Utilizing a false discovery rate of <0.05, we found that 2126 genes were differentially regulated in AD hippocampal neurons compared to controls when combining the two studies (Additional file 1: Table S1). We next wanted to compare the DEGs with genes that have been identified as expressed in CA1 hippocampal neurons. Using the rat CA1 hippocampal cell body transcriptome identified by Cajigas et al. [20], 74.5% of the DEGs found in the studies we examined from aged brains are indeed known to be expressed in CA1 hippocampal neurons, confirming the efficacy of the microdissection.

To achieve a functional understanding of the DEGs, we then probed their biological function using the Ingenuity Pathway Analysis tool (IPA) which curates biological function based on annotated ontologies. While analysis did not reveal any neuron/brain specific functions, we found an over representation of terms suggesting dysfunctions in core homeostatic pathways: protein synthesis, repression of RNA, and cell death/apoptosis (Table 2). Along with all cell types, regulation of gene expression, the synthesis of proteins and the control of cell death play a detrimental roll in neuron health, survival, and overall brain function. The genes represented by the functional annotation Apoptosis are also found with the genes in the Cell death annotation, and combining these lists resulting in a total of 329 unique genes participating in these homeostatic processes (Additional file 2: Table S2). This led us to the hypothesis that translation, transcription and cell death/apoptosis are mechanisms through which AD compromises neuronal integrity. For this reason we chose to further investigate these genes to identify key specific genes to these homeostatic pathways and AD.

**Table 2:** Biologic functional analysis of DEGs

**Category**	**Functions annotation**	**P**-**value**	**Predicted activation state**	**Regulation z**-**score**	**Number of genes**
Gene expression	Repression of RNA	9.19E-03	Increased	2.617	17
Cell death	Cell death	8.85E-03	Increased	2.234	291
Cell death	Apoptosis	2.48E-02	Increased	2.168	213
Protein synthesis	Synthesis of protein	4.92E-05	Decreased	−2.064	40

IPA analysis of functions (p < 0.05, z-score > |2|), with predicted activation state.

### Biological network generation of homeostatic process genes utilizing protein-protein interactions

**Figure 2:**
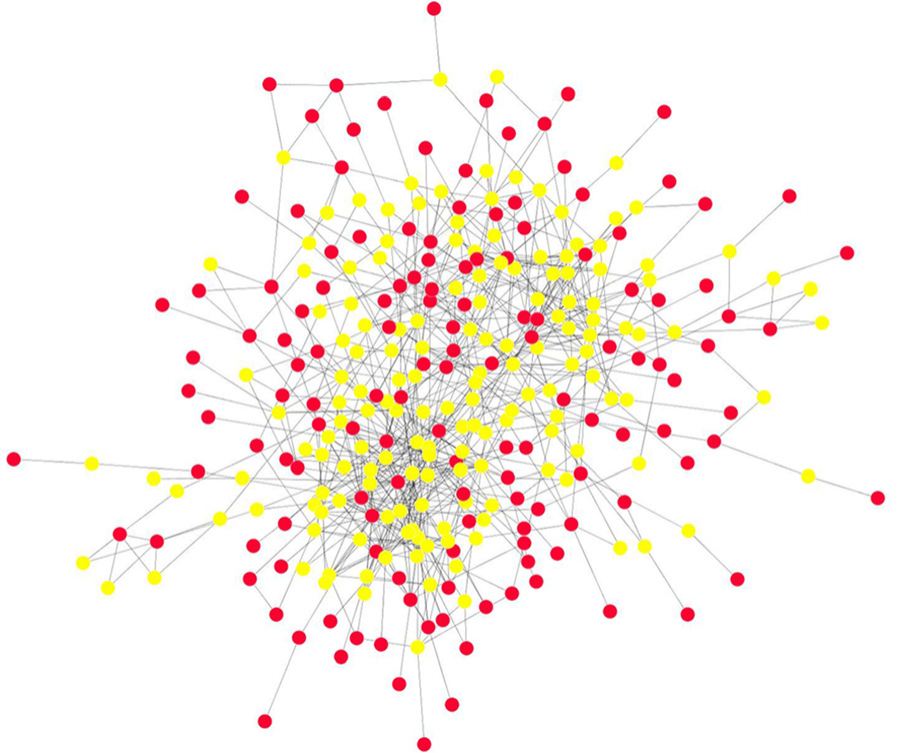
**Protein**-**protein interaction networks.** The network (biological function) is made up of 1^st^ and 2^nd^ degree protein-protein interactions. Red nodes represent the proteins encoded by genes identified as DEG belonging to the homeostatic pathways, yellow nodes represents proteins added as interacting partners.

We next wanted to find which of these genes interacted with each other directly and through other partners, thus building on the gene list by incorporating additional interacting partners that may not have been identified as significantly differentially expressed. Because the protein products of genes do not act alone but rather cooperate with other proteins to perform a function, we assessed the protein-protein interactions (PPIs) of the focal 329 genes. For this we used Genes2Networks, which integrates the data contained in multiple interaction network datasets, enables the user to determine the path length (degree of interaction), and incorporates possible interacting proteins which are not part of the user’s original input list. We chose to explore 1^st^ (direct) and 2^nd^ (through another protein) degree PPIs among the protein products of the focal genes to gain complexity without losing specificity of the overall network. The resulting PPI network consisted of 305 nodes and 727 edges (Figure 2). PPI networks hold an immense amount of information not only about individual proteins and pathways but also on directional flow and interconnection of biological functions. In order to understand key protein players within the network, we next employed graph theory modeling for exploration of the large number of proteins within the network.

### Identification of central AD genes through graph theory analysis

We then set out to identify the key genes crucial to the mechanism of the overall interactome of these proteins. Communication within networks is key to their functions as a whole. PPI networks can form hubs and complexes, or show enzymatic relationships. To better understand critical nodes within our PPI networks, we used graph theory for centrality modeling, specifically closeness, eccentricity, and radiality. These measure the network’s topology and use individual node proximities in finding highly central nodes. Being highly central to communication of the network highlights the importance of that node in the overall functions characterized [21]. Closeness, eccentricity and radiality are similar but achieved differently mathematically; with radiality and closeness being quite similar with the exception that radiality is based on individual network diameter whereas closeness is not. Each computes the shortest paths between single proteins and all other proteins in the network. By using these three similar yet differently calculated centralities, we can compare node placement between the measurements. This will identify highly central nodes for further investigation.

**Figure 3:**
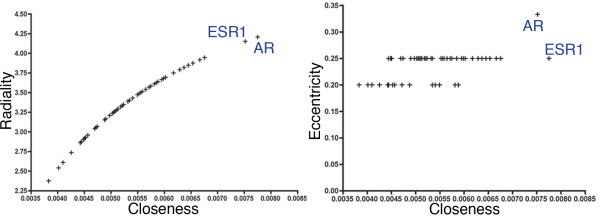
**Radiality**, **eccentricity**, **and closeness.** Closeness (X-axis) is plotted versus Radiality (left) and Eccentricity (right) on the Y-axis. Each axis represents the range of above average values for the centrality measures. The positions of ESR1 and AR are indicated.

We utilized CentiScaPe to calculate node centrality values. We restricted our interest into nodes with above average values when comparing closeness, radiality, and eccentricity, terming them as above average nodes (Figure 3). As expected for the networks, closeness and radiality had a linear relationship due to their similar calculations. When comparing closeness and eccentricity, the androgen receptor (AR) and the estrogen receptor alpha (ESR1) stand out as the top two nodes within the network. ESR1 has a greater eccentricity value than AR. This places more proteins in ESR1’s proximity suggesting greater role in dictating functional directions within the network. AR has a greater closeness than ESR1 placing AR in proximity of more proteins than ESR1. This suggests AR works more through competition to achieve specific functions. Indeed sex steroids have been implication in AD pathogenesis [22].

### Isolating dense network regions of key AD genes through cluster analysis

**Figure 4:**
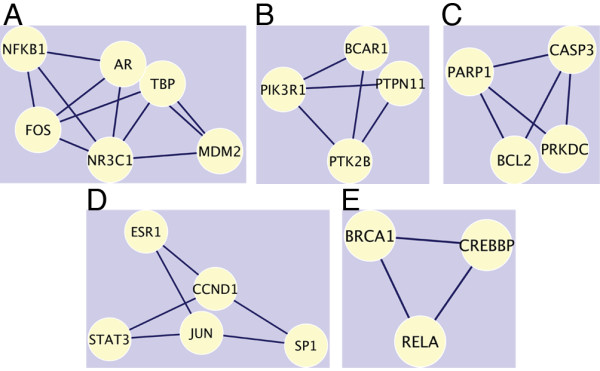
**MCODE complexes.** Clusters **A**, **D**, and **E** contain transcription factors, with AR present in Cluster **A** and ESR1 in Cluster **D**. Clusters **B** and **C** contain kinases and ligases but not transcription factors. Yellow nodes represent proteins, indicated by their symbols, and interconnections by black edges.

After graph theory analysis, we wanted to better understand the relationships between the PPI network proteins that were above average for closeness, radiality, and eccentricity; the 73 genes encoding these proteins were termed highly central genes (Additional file 3: Table S3). Before understanding the interconnection between these genes, we once again examined whether these genes were reported to be expressed in CA1 hippocampal neurons. Indeed 90.4% of these above average, highly central genes are expressed by these cells [20]. Highly interconnected, dense areas within a network can denote protein clusters or parts of pathways. Using MCODE, we identified five of these dense regions within the network (Figure 4). The first dense region (Figure 4A) contains AR along with transcription factors, including the glucocorticoid receptor (NR3C1), which belongs to the same protein subfamily and group as AR. With the other proteins, such as FOS and NFKB1, this cluster represents a complex of transcription factors. Similarly ESR1 was found in the fourth cluster with other transcription factors including SP1, JUN and STAT3 (Figure 4D). The fifth cluster also identifies transcription factors and a regulator, CREBBP, RELA, and BRCA1 (Figure 4E). The second and third clusters (Figure 4B,C) do not contain any transcription factors, but rather kinases and ligases that carry out signal and degradation pathways. Taken together these clusters indicate that the original first list of genes/proteins contain transcription, degradation, and signaling hubs that regulate the transcription, translation, and cell death/apoptosis in AD.

### Corroboration of the selected genes’ involvement in AD pathology

We predicted that the proteins produced during the graph theory and cluster analyses would be integral for AD dysfunction. To test this, the proteins above centrality averages in the graph theory assessment were used as input for three independent systems biology tools: two propriety databases (IPA and MetaCore), and the publically available database DAVID. These genes were examined for enrichment in “diseases and disorders”. Many of the genes had links to cancer for all three databases due to the high amount of experimental data on cancer curated within many databases, still in each case these genes were significantly enriched in pathways for neurodegenerative disorders, specifically AD (Table 3). First for IPA, AD was found to be enriched, as well as, at lower levels of significance, Parkinson’s Disease and Huntington’s Disease. The second database, MetaCore, also identified gene enriched for neurodegenerative disease, dementia, and AD, and at a much lower significance Parkinson’s Disease. Finally we used DAVID to look for disease ontology enrichment and found AD, and at a lower level of significance, Parkinson’s Disease. In all three databases AD ontology had a greater statistical significance than other neurodegenerative disorders such as Parkinson’s or Huntington’s Disease. While there are indeed similarities in pathways activated to result in neuronal damage and death in neurodegenerative diseases, the greater significance found for AD validates the role of these genes in the deregulation of transcription, translation, and cell death/apoptosis in CA1 hippocampal neurons affected by AD.

**Table 3:** Biologic functional analysis of highly central genes

**Bioinformatics Platform**	**Disease**	**p**-**value**
IPA	Alzheimer's Disease	4.40E-08
	Parkinson's Disease	2.88E-04
	Huntington's Disease	1.28E-03
MetaCore	Neurodegenerative Diseases	4.20E-25
	Dementia	5.98E-24
	Alzheimer's Disease	3.30E-23
	Parkinson's Disease	2.15E-13
DAVID	Alzheimer's Disease	1.40E-03
	Parkinson's Disease	1.10E-02

IPA, MetaCore, and DAVID analysis of neurological disease enrichment. The diseases identified were based on the 73 genes that are highly central in the PPI network.

## Discussion

The data in this study highlights the ability of meta-analysis and systems biology approach to help unravel the complexity of AD. When looking specifically at biological functions, AD disrupts protein synthesis, cell death/apoptosis, and gene expression through the repression of RNA. These processes are essential for the health of neurons. A recent meta-analysis study for AD was conducting using 100 publically available microarray datasets from hippocampus brain samples [23]. While this paper focused more on AD progression, similar changes were identified in the cellular functions of protein and gene expression regulations. Taken together with our study this suggests that AD affects very important yet basic cellular, homeostatic processes such as the ones identified here: transcription, translation, and cell death/apoptosis.

While men and women express both AR and ESR1 receptors, the levels of their ligands, testosterone and estrogen, separate the sexes. ESR1 and estrogen work to promote female characteristics and reproductive maintenance. Besides these huge defining attributes, estrogen works in a variety of genomic and non-genomic ways. AR and testosterone are important in male development and like estrogens work outside of a sexual role. Both estrogen and testosterone demonstrate neuroprotective qualities and have been implicated in AD development and progression. Aging causes a decrease in testosterone and estrogen levels, which several studies have linked to an increase incidences of AD.

Many studies focusing on the relationship between estrogen, AD and the female brain have found that the increase in AD in women is not due to their increased lifespan [24-33]; the female brain appears to be more vulnerable to AD pathology. This is also found in the Tg2576, APPswexPS1 and 3xTg-AD triple AD mouse models when females are age-matched to males. Females of all three models display higher Aβ load burden and plaque quantity as compare to the male counterparts. [34-36] Neuroprotective properties come from estrogen’s connection with the Bcl2 family in apoptotic pathways [37-41], involved in excitotoxcity [42-47], inflammation [48,49], and oxidative stress [50,51].

The role of menopause and subsequent Hormone or Estrogen Replacement therapy (HRT or ERT) in AD has been part of the experimental discussion. The decreased level of estrogen resulting from menopause is thought to cause a woman’s increased vulnerability to AD. Many female AD patients due indeed have lower than usual estrogen levels and studies have shown that low estrogen increases incidence of AD. Despite these links between estrogen and AD, the results are mixed on the benefits of HRT/ERT for AD development and progression. Most studies suggest that HRT/ERT is beneficial if a long-term regime is adhered to [52-59] and decrease the risk of cognitive dysfunction [60-63] while the other studies are inconclusive on overall benefit [64-73]. The time at which HRT is administered may also affect AD risk. HRT five or more years after menopause may negate its protective role in reducing the risk of developing AD [74]. The truth behind HRT’s role in AD may not be discovered until HRT/ERT itself is properly understood, prescribed, and used.

Additional studies linking ESR1 to AD have examined changes in its nuclear versus cytoplasmic subcellular distribution in the hippocampus [75-77], as well as membrane localization leading to estrogen-induced activation of hippocampal glutamate receptors in the absence of glutamate [78]. Furthermore differences between ESR1 alleles correlates with an increased risk for AD in women with Down syndrome [79]. Finally, in neural cell lines, ESR1 has been linked to neuroprotection [80]. Through the ESR1 specific agonist, propylpyrazole, the receptor’s activity has been shown to protect against Aβ accumulation [81] and from glutamate excitotoxicity through ERK signaling and upregulation of Bcl-2 at the gene level [82,83].

Aging men are not exempt from the connection between AD and sex hormones. As stated earlier both ESR1 and AR are both implicated in AD and it’s progression, but the extent of each receptor’s role remains unclear [84,85]. A similar increased AD risk is associated with low testosterone levels in men. Like estrogen, testosterone has been shown to regulate levels of Aβ. However, the blocking of testosterone to estrogen converting enzyme, aromatase, in one study attributes neuroprotection with estrogen alone [86]. However testosterone and AR play their own role in neuroprotection from Aβ accumulation. The male population presents the opposite model as a posed to the female population; sufferers of prostate cancer undergo anti-androgen therapy causing a reduction in testosterone levels. Studies in these patients have shown increased levels of plasma Aβ [87,88].

AR and ESR1 are known to have functional importance in AD; this was a topic of discussion well before our study or the studies used within our study were conceived. Our meta-analysis and systems biology analyses were capable of identifying these “chains” of AD changing genes surrounding AR and ESR1. Our findings integrate well into and help fill current gaps in our knowledge on AD.

By focusing on differentially expressed genes, their protein-binding partners of their products, and utilizing graph theory, we were able to broaden our knowledge of AD’s pathogenesis. Proteins/genes do not live in a vacuum and so changes in expression affects the ability of other proteins/genes to function. Available levels of proteins can cause competition between binding partners, which then can in turn shunt pathways to be activated, inhibited or perhaps balance out. The clusters for each sub network are based on highly interconnected areas, which represent protein complexes. The level and variety of proteins comprising the complex can give the individual complex a “fingerprint” that elicits various functions.

## Conclusions

Meta-analysis and systems biology approaches allowed us a unique view of hippocampal neuron-specific transcriptomic analysis in AD. By using meta-analysis combining data from independent studies, we were able to identify deregulation of genes that participate in transcription, translation, and cell death/apoptosis in CA1 hippocampal neurons from AD patients. Further investigation of these genes and their interactions led to the identification of genes important to the overall mechanism of the deregulated homeostatic processes. The two centrally highest genes, AR and ESR1, and their role in AD pathology are under examination clinically and in experimental models. Use of a final method based on cluster density, reduced our focus to a smaller set of genes. Based on three independent bioinformatics tools, these genes are enriched for processes involved in AD pathophysiology. Taken together with our initial findings these genes play a role in AD through dysregulation of the basic homeostatic processes of transcription, translation, and cell death/apoptosis.

## Methods

### GEO data retrieval, microarray normalization and ensemble mapping

The Gene Expression Omnibus (GEO) (http://www.ncbi.nlm.nih.gov/geo/) is a public repository of various genetic high through put data sources. Microarray data sets for different chips types are deposited with extensive experimental design and information and with normalized and/or raw data. We set our experimental criteria specific to postmortem human hippocampal samples analyzed using an Affymetrix platform. Our search obtained two GEO data sets, GSE5281 and GSE28146 (we only utilized the arrays from severe AD patients from this study), which contained CA1 region specific individual hippocampal neurons data. The appropriate control and AD raw CEL files were downloaded from the GEO site. Next the CEL files were read into the R programming console using the affy package [89] (this and other R-based tools were obtained from the open source Bioconductor bioinformatics software, http://www.bioconductor.org). In the uploading process CDF files were associated with the microarrays through the GeneMapper package. Downloaded from GATExplorer [89-91] GeneMapper (http://bioinfow.dep.usal.es/xgate/mapping/mapping.php) includes the sets of unambiguous probes that map to each specific Ensemble Gene ID (ENSG ID). Also through affy package, the intensity files were normalized using Robust Multichip Average, RMA. A three-step process, RMA performs a background adjustment, quantile normalization and final summarization [92].

### Ethics

The data utilized were obtained from a research repository databank (GEO), involves decedents, contains no personal identifiers, and the authors had no role in the collection or storage of these data.

### RankProd

The R RankProd package contains functions for differential gene expression analysis of microarrays based on a non-parametric statistic [93]. RankProd identifies genes that are consistently highly ranked amount a list of genes. Since the method exploits the rank of genes not the actual expression value, it can be flexibly applied to many different questions, such as identifying genes.

It assumes that under the null hypothesis the order of genes are random and statistically probabilities are based on the probability of a particular ranking. Rank product is the multiplication of these probabilities. RankProd produces a list of up- or down- regulated genes with false discovery rate (FDR). RankProd also has the ability to combine data sets from different origins into a single meta-analysis to increase the power of the identification [93].

### Ingenuity pathway analysis

IPA is commercially available software (Ingenuity Systems, Inc., Redwood City, CA) for several types of analysis and is popular in a variety of biological fields/studies. IPA utilizes a large, well-designed knowledge base and enables functional, canonical pathway and network analysis. IPA uses its knowledge base to better understand how the data fit with the curated functional, canonical pathway, and interaction network information. We utilized the Functional Analysis tool to identify the biological functions and/or diseases that were most significant to the data set. Molecules from the dataset were associated with biological functions and/or diseases in the Ingenuity Knowledge Base were considered for the analysis. Right-tailed Fisher’s exact test was used to calculate a p-value determining the probability that each biological function and/or disease assigned to that data set is due to chance alone.

### Genes2Networks

A publically available bioinformatics database, Genes2Networks (http://actin.pharm.mssm.edu/genes2networks/) is hub of databases used to find relationships between genes and proteins from seed lists [94]. Predictions of genes or proteins that may play crucial roles in pathways or protein complexes are supplemented to the seed list. Gene2Networks calculates a Z statistic using a binomial proportions test on the significance of a supplemented protein in the output sub network.

### Cytoscape plugins

Cytoscape, a popular publically available bioinformatics package (http://www.cytoscape.org), represents networks, with biological entities as nodes and biological interactions as edges between nodes [95]. Plugins are designed to run several types of analysis. Before plugins are applied all satellite networks are removed leaving a single interconnected network of nodes. This study uses two such plugins, CentiScaPe and MCODE.

### CentiScaPe

CentiScaPe is an interface to analyze topology of protein-protein interaction networks [96]. CentiScaPe uses a variety of graph theory centrality measurements to determine and develop sub networks. In this study we used the following:

#### Eccentricity

Eccentricity computes the shortest paths between a single node and all other nodes in the graph. Next the longest shortest path is chosen and the reciprocal is taken. Higher value represent nodes that have the shortest paths, meaning all other nodes are in its proximity. All other proteins within the network easily reach proteins with high eccentricity. Thus, a protein with high eccentricity dictates functional directions, but also is subjected to functional control by binding partners. On the opposite end, a low eccentricity suggests a peripheral functional role.

#### Closeness

Closeness computes the shortest path between a single node and all other nodes. The summation is taken of the shortest paths and the reciprocal is taken. Nodes with high closeness are in close proximity to all other nodes in the network. If the closeness measure is low, all other nodes are distant from this node. Closeness measurements can also reflect few nodes that are very close or distant from a specific node. Therefore closeness is not specific to the nature of the node couples and should be compared with eccentricity and radiality. In relation to PPI networks, closeness can represent functionally and points of competition between proteins.

#### Radiality

Radiality is calculated similarly to closeness, but subtracts the diameter of the graph from each path. This value is then summated and finally divided by the number of nodes minus 1. Short paths have high radiality values where as long paths have low values. With respect to the diameter high radiality nodes are closer to other nodes. In PPI networks radiality can represent functional relevancy between a single protein and other proteins and functional directional control.

### MCODE

Another plugin used Molecular Complex Detection, MCODE, formulates clusters within a network [97]. MCODE, a theoretic clustering algorithm, identifies densely connected areas in large protein-protein interaction networks that could suggest protein complexes. MCODE is built on vertex weighting by local neighborhood density and outward crossing from a locally dense protein isolating the dense regions according to user specific parameters. [97] MCODEv1.32 was used for cluster identification among the proteins with the two sub networks.

### MetaCore

MetaCore is commercially available software (GeneGo, Thompson Reuters, New York, NY) for functional analysis of high throughput data. For this study we focused on using the Disease (by biomarker) analysis. MetaCore bases disease ontology on classifications in Medical Subject headings. Each disease has a corresponding biomarker gene or sets of genes and p-value statistic based on the probability of a random intersection of two different gene sets. The p-value of the intersection between an experimental gene and ontology is considered as a measure of relevance of said ontology to the experimental dataset.

### The Database for Annotation, Visualization and Integrated Discovery (DAVID)

DAVID is a publically available database (http://david.abcc.ncifcrf.gov) that offers a comprehensive set of functional annotation tools to recognize biological meaning behind list from high through put experiments. For this study we focused on using the disease information in the Genetic Association Database curated within DAVID’s functional analysis. The statistics used for predicted disease ontologies are p-values using Fisher’s exact test.. Each statistical measure corresponds to the probability of one or more genes overlapping with the predicted disease ontology.

## Competing interests

The authors declare that they have no competing interests.

## Authors’ contributions

The study was designed by JMW and HSF. Analysis was carried out by JMW, and interpreted by JMW and HSF. The paper was written by JMW, edited by HSF, and approved by both authors.

## Supplementary Material

Additional file 1: Table S1 All differentially expressed genes (FDR<0.05) between AD and control. Click here for file

Additional file 2: Table S2 Differentially expressed genes identifying enriched homostatic processes through IPA analysis. Click here for file

Additional file 3: Table S3 Highly central genes identified through graph theory analysis. Click here for file
